# A helping HAND: therapeutic potential of MAGL inhibition against HIV-1-associated neuroinflammation

**DOI:** 10.3389/fimmu.2024.1374301

**Published:** 2024-05-21

**Authors:** Alexis F. League, Barkha J. Yadav-Samudrala, Ramya Kolagani, Calista A. Cline, Ian R. Jacobs, Jonathan Manke, Micah J. Niphakis, Benjamin F. Cravatt, Aron H. Lichtman, Bogna M. Ignatowska-Jankowska, Sylvia Fitting

**Affiliations:** ^1^ Department of Psychology and Neuroscience, University of North Carolina at Chapel Hill, Chapel Hill, NC, United States; ^2^ Department of Pharmaceutical Sciences, Skaggs School of Pharmacy and Pharmaceutical Sciences, University of Colorado Anschutz Medical Campus, Aurora, CO, United States; ^3^ Department of Chemistry, Scripps Research, La Jolla, CA, United States; ^4^ Department of Pharmacology and Toxicology, Virginia Commonwealth University, Richmond, VA, United States; ^5^ Neuronal Rhythms in Movement Unit, Okinawa Institute of Science and Technology, Okinawa, Japan

**Keywords:** HIV-1, inflammation, transactivator of transcription (Tat), endocannabinoids, HETE, monoacylglycerol lipase (MAGL)

## Abstract

**Background:**

Human immunodeficiency virus (HIV) affects nearly 40 million people globally, with roughly 80% of all people living with HIV receiving antiretroviral therapy. Antiretroviral treatment suppresses viral load in peripheral tissues but does not effectively penetrate the blood-brain barrier. Thus, viral reservoirs persist in the central nervous system and continue to produce low levels of inflammatory factors and early viral proteins, including the transactivator of transcription (Tat). HIV Tat is known to contribute to chronic neuroinflammation and synaptodendritic damage, which is associated with the development of cognitive, motor, and/or mood problems, collectively known as HIV-associated neurocognitive disorders (HAND). Cannabinoid anti-inflammatory effects are well documented, but therapeutic utility of cannabis remains limited due to its psychotropic effects, including alterations within brain regions encoding reward processing and motivation, such as the nucleus accumbens. Alternatively, inhibiting monoacylglycerol lipase (MAGL) has demonstrated therapeutic potential through interactions with the endocannabinoid system.

**Methods:**

The present study utilized a reward-related operant behavioral task to quantify motivated behavior in female Tat transgenic mice treated with vehicle or MAGL inhibitor MJN110 (1 mg/kg). Brain tissue was collected to assess dendritic injury and neuroinflammatory profiles, including dendritic microtubule-associated protein (MAP2ab) intensity, microglia density, microglia morphology, astrocyte density, astrocytic interleukin-1ß (IL-1ß) colocalization, and various lipid mediators.

**Results:**

No significant behavioral differences were observed; however, MJN110 protected against Tat-induced dendritic injury by significantly upregulating MAP2ab intensity in the nucleus accumbens and in the infralimbic cortex of Tat(+) mice. No or only minor effects were noted for Iba-1^+^ microglia density and/or microglia morphology. Further, Tat increased GFAP^+^ astrocyte density in the infralimbic cortex and GFAP^+^ astrocytic IL-1ß colocalization in the nucleus accumbens, with MJN110 significantly reducing these measures in Tat(+) subjects. Lastly, selected HETE-related inflammatory lipid mediators in the striatum were downregulated by chronic MJN110 treatment.

**Conclusions:**

These findings demonstrate anti-inflammatory and neuroprotective properties of MJN110 without cannabimimetic behavioral effects and suggest a promising alternative to cannabis for managing neuroinflammation.

## 1 Introduction

Human immunodeficiency virus (HIV) remains a major global public health issue, with 39.0 million (33.1-45.7 million) people living with HIV (PLWH) worldwide at the end of 2022, out of which 76% (65-89%) received combination antiretroviral therapy (cART) (1). The overall prevalence rate of HIV-1 infection is higher among adult women than men, with 15% more women living with HIV-1 relative to men in 2019 (2). Importantly, HIV-1 affects women differently than men, with greater immune activation observed in women despite lower overall viral load (3). Given the historical underrepresentation of females across human and animal models of HIV infection, the present study centers specifically on females to better characterize the effects of HIV and novel intervention strategies in this statistically underrepresented population.

While cART successfully reduces viral load in peripheral tissues and significantly improves life expectancy of PLWH (4–6), these treatments are largely incapable of effectively crossing the blood-brain barrier (BBB) to suppress replication of viral proteins, such as transactivator of transcription (Tat), which enters the central nervous system (CNS) within two weeks of infection both directly and through peripherally infected monocytes and macrophages (7–10). Secretion and synthesis of viral protein reservoirs persist in the CNS, where they alter the cellular environment, contributing to chronic neuropathy and HIV-associated neurocognitive disorders (HAND), even in patients actively undergoing treatment (7). Underlying HAND are several dysfunctional immunomodulatory mechanisms within the CNS, including persistent inflammation and immune activation (11–14), which ultimately lead to dendritic injury and damage to neurons (15–17). Notably, cART itself also contributes to both neuroinflammation and altered neuronal connectivity with prolonged exposure (18, 19). In preclinical animal studies, the HIV Tat transgenic mouse model is a well-established model for neuroHIV since their neuropathology and behavioral deficits tend to mirror those observed in cART-treated PLWH with HAND (20–22). These include structural abnormalities in neurons/dendrites, such as reduced spine density and changes in synaptic proteins (21–23), disrupted frontostriatal circuitry (24), and glial abnormalities including microglial activation and micro/astrogliosis (20, 21, 25). Further, these mice and related neuroHIV rodent models also develop changes in learning/memory, motor activity, and motivated behaviors (24, 26–30) relevant for cART-treated PLWH. As dopaminergic neurocircuitry is highly susceptible to disruption by HIV proteins, including HIV Tat (29, 31–34), motivational alterations remain problematic neurobehavioral manifestations in cART-treated PLWH, including apathy (35, 36) which parallels lack of motivation.

The endogenous cannabinoid (endocannabinoid) system is a critical line of defense against neurodegenerative and inflammatory conditions (37). It is well established that endocannabinoids, such as 2-arachidonoylglycerol (2-AG) and N-arachidonoylethanolamine (AEA) upregulate in certain disorders (e.g., Parkinson’s disease, Alzheimer’s disease, multiple sclerosis) and reduce or abolish unwanted effects of these disorders or slow their progression (38, 39). Recent work has demonstrated therapeutic potential of inhibiting monoacylglycerol lipase (MAGL), the primary enzyme that hydrolyzes 2-AG. 2-AG, is an endogenous ligand at neuronal cannabinoid type-1 receptors (CB_1_R) and, importantly, also an agonist at cannabinoid type-2 receptors (CB_2_R) in immune cells throughout the brain and body (40). Activation of CB_2_R has shown protective properties against neuroinflammation and neurodegenerative disorders (41). MAGL-focused strategies are of great interest because targeting enzymatic breakdown is more beneficial relative to phytocannabinoids, as 2-AG tone is affected locally on-demand where its breakdown is dysregulated, thus reducing off-target effects relative to exogenous agonists (42). Because the downstream products of 2-AG hydrolysis, including arachidonic acid (AA), are themselves broken down into proinflammatory eicosanoid lipid mediators such as prostaglandins, hydroxyeicosatetraenoic acids (HETEs), and epoxyeicosatrienoic acids (EETs), inhibiting 2-AG breakdown also stands to reduce proinflammatory processes (43, 44).

Previous studies have demonstrated the neuroprotective properties of MAGL inhibition across numerous models of brain damage and inflammation (45–48), including suppressed astrocytic and microglial activation in models of Alzheimer’s disease (49, 50) and reduced HIV-1 envelope glycoprotein (gp120)-associated synapse loss and IL-1β (51). Further, as 2-AG plays a significant role in modulating mesolimbic dopamine release and associated behaviors (52), inhibiting 2-AG degradation by targeting MAGL has been shown to enhance dopamine signaling, reward-related behavior, and motivation with the MAGL inhibitor JZL184 (53, 54). While JZL184 targets 2-AG more potently and selectively (55) compared to earlier-generation MAGL inhibitors such as URB602 (56), studies have shown desensitization of CB_1_R, which can contribute to physical dependency (57), as well as cross-reactivity with other serine hydrolases including α/β hydrolase domain (ABHD) (55). Newer-generation MAGL inhibitors such as MJN110 confer greater 2-AG selectivity with adequate potency to effectively inhibit MAGL *in vivo* at doses as low as 1 mg/kg (58). MJN110, in particular has shown antinociceptive, anti-inflammatory, and neurorestorative effects previously (59, 60). While MJN110 has also previously been shown to increase reward-directed behavior, this observation was specific to doses of 5 and 10 mg/kg (61). Given these findings, we were interested in characterizing whether Tat or lower-dose MJN110 may affect motivated behavior in the present model.

Thus, the present study used the HIV-1 Tat transgenic mouse model to investigate the chronic effects of MAGL inhibitor MJN110 (1 mg/kg) on reward-related motivated behavior. Immunohistochemical and lipid mediator analyses were conducted to assess potential anti-inflammatory and neuroprotective effects of chronic MJN110 exposure against Tat-induced toxicity on the CNS system, with focusing on the ventral striatum (nucleus accumbens) and infralimbic cortex. It is hypothesized that MJN110 will reduce Tat-driven dysregulation in motivated behavior and exert protective effects against Tat-driven neuroinflammation and neuronal injury.

## 2 Materials and methods

### 2.1 Subjects

Brain-specific, astrocyte-driven HIV-1 Tat_1-86_ expression was induced in a Tet-on system using doxycycline in transgenic female mice (*N* = 34) as previously described (62). Briefly, mice were developed on a hybrid C57BL/6J background wherein Tat(+) subjects expressed both glial fibrillary acidic protein (GFAP)-reverse tetracycline-controlled transactivator (rtTa) and tetracycline-responsive element (TRE)-tat genes, while control Tat(−) subjects expressed only the GFAP-rtTA gene (20). Genotyping was performed 7-10 days post-weaning to confirm Tat transgene expression. Subjects at ~ 4 months of age (age range: 3-6 months) were provided ad libitum access to water and doxycycline-containing chow (6 mg/g; Envigo, NJ, USA; #TD.09282) for three months preceding and throughout behavioral assays to establish and maintain a chronic exposure model (63). Note that estrous cycle was not monitored due to the concern that stress associated with daily vaginal lavage could confound our dependent measures (64). Subjects were maintained on a 12-hour reversed light/dark cycle, and behavioral data were collected during the dark phase only. Experimenters were blind to genotype throughout data collection. All procedures were conducted in strict accordance with the ethical guidelines outlined in the NIH Guide for the Care and Use of Laboratory Animals (NIH Publication No. 85-23) and approved by the Institutional Animal Care and Use Committee (IACUC, Protocol#: 23-056.0) at the University of North Carolina at Chapel Hill.

### 2.2 Drug treatment

Drug assignment (MJN110/vehicle) was randomized and counterbalanced across genotype groups [Tat(−) and Tat(+) mice] to yield four total genotype/drug groups with 8-9 subjects each [Tat(−) Vehicle, *n* = 9; Tat(−) MJN110, *n* = 8; Tat(+) Vehicle, *n* = 8; Tat(+) MJN110, *n* = 9]. MJN110 (1 mg/kg) was dissolved in a vehicle solution containing a 1:1:18 ratio of Kolliphor (Sigma-Aldrich, #C5135), ethanol (Decon Laboratories, #64174), and 0.9% sodium chloride (Braun Medical, #J8K944) as described previously (58). Mice assigned to the vehicle group received the vehicle solution without the MJN110 drug. MJN110 dosage was chosen based on prior studies which demonstrated 1 mg/kg oral MJN110 administration was sufficient to partially block MAGL in the brain, precluding tolerance development which occurs with full blockade via CB_1_R desensitization (58). Additionally, subcutaneous 1 mg/kg MJN110 injections 2 hours prior to an intraperitoneal acid injection showed a prominent antinociceptive profile that tended to be greater in female rats compared to males (65). Subcutaneous injections (10 µL/g) were performed daily for two weeks preceding and throughout behavioral assessments (3-22 days, depending on animal’s performance). This method of delivery was chosen to most closely mimic oral administration and preclude complications associated with intraperitoneal injections (e.g., lower full-blockade dose, faster absorption, and greater potential for intestinal irritation) (66). Injections were administered at the same time each day, approximately two hours before data collection to ensure maximal effect during the testing window (58). Subject weight was recorded daily for dosing accuracy and to ensure body mass remained stable across the treatment time course.

### 2.3 Reward-related operant behavioral task

#### 2.3.1 Experimental design

**Figure 1:**
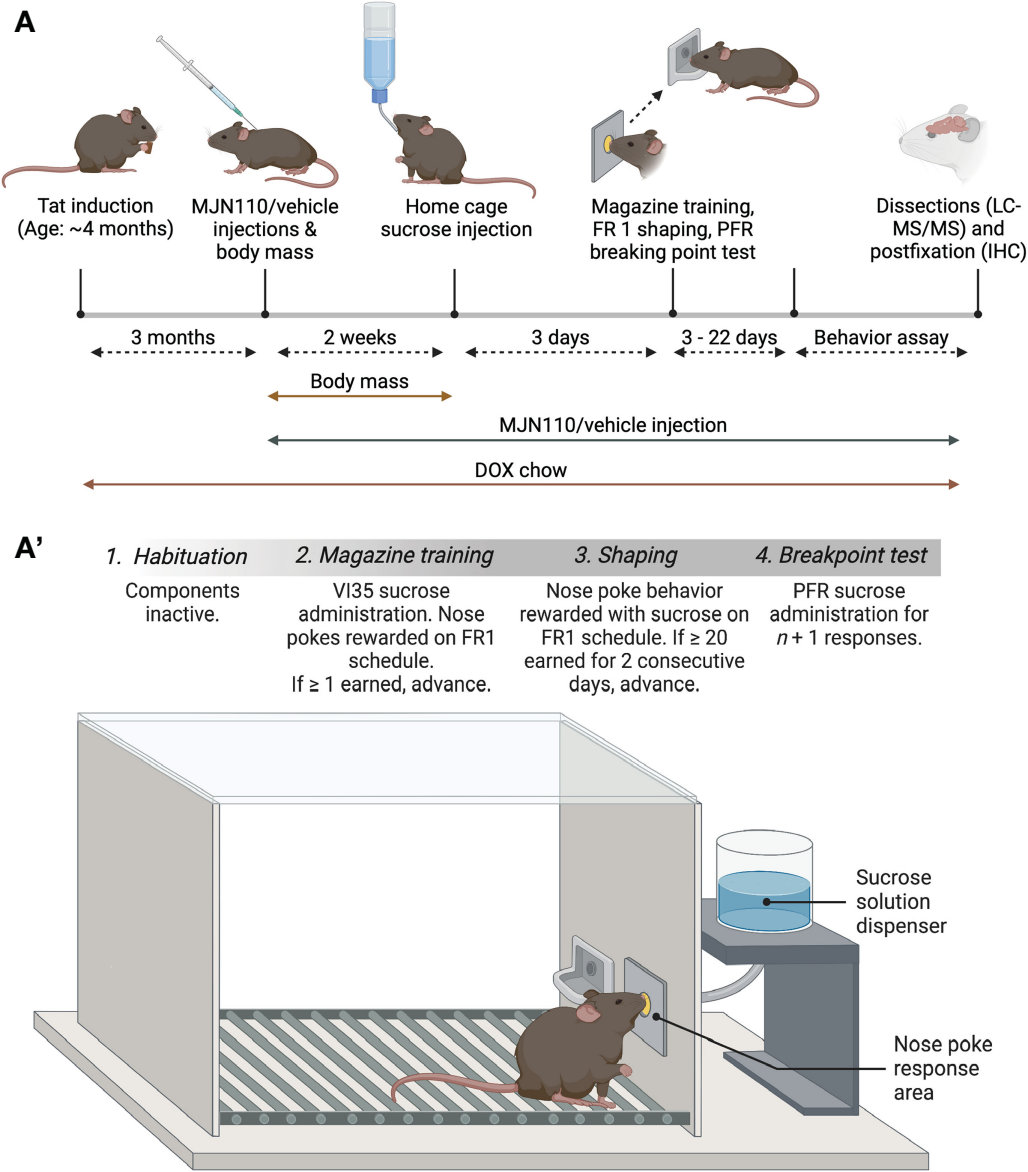
Schematic representation of the experimental timeline **(A)** and the behavioral assay **(A’)**. **(A)** After mice received DOX-containing chow for 3 months, mice were subcutaneously injected with vehicle/MJN110 for 2 weeks. DOX food and drug injections continued throughout the experimental study. Body mass was recorded daily when drug injections started. After 2 weeks of drug injections, subjects were habituated for 3 days in their home cage to the 25% sucrose solution (w/v). The behavioral assay started the next day and lasted between 3-22 days depending on animal’s performance **(A’)**. Figure created with BioRender.com. DOX, doxycycline; FR1, fixed-ratio 1; PFR, progressive fixed-ratio; VI35, variable-interval schedule (30-45 seconds).

Subjects were trained to nose poke for 25% sucrose solution (w/v), similar in concentration to that used in previous studies (67, 68). The procedural design described herein was adapted from Nam and colleagues (69) (**Figure 1**). Subjects first underwent habituation and magazine training to acclimate to test procedures and stimuli. After successfully making the association between nose poke behaviors and sucrose availability and learning to respond consistently, subjects advanced to a motivation test, which assessed how many nose poke behaviors they would perform to earn a single sucrose reward.

#### 2.3.2 Apparatus

Behavior was recorded in operant chambers (MED Associates, #ENV-307W) containing a nose poke response port and adjacent reward dispenser (MED Associates, #ENV-302W-S). To reduce potential visual and auditory distractions, operant chambers were contained within sound-attenuating cubicles providing 80 dB white noise (MED Associates, #ENV-022MD). MED-PC computer interface software was used to automate sessions and record behavior. To ensure testing environment consistency across training and test phases, subjects were randomly assigned an operant chamber in which they were tested throughout all sessions.

#### 2.3.3 Habituation

Subjects were habituated to experimenter handling for two weeks preceding behavioral training. Three days before operant chamber habituation, water in home cages was replaced with 10% sucrose (w/v) on days 1 and 2, then 20% sucrose (w/v) on day 3. Sucrose concentration was increased across the habituation period to allow subjects time to adapt and develop consummatory behaviors towards the solution. To increase reward salience for magazine training, sucrose solution was replaced with water on the day of test environment habituation (day 4), wherein subjects were exposed to operant chambers and white noise for 30 minutes, with nose poke response ports and reward dispensing components inactive.

#### 2.3.4 Magazine training

The following day, subjects underwent a one-hour training session wherein all operant chamber components were active. During this phase of training, 25% sucrose solution (w/v) was made available for 20 seconds at a time on a variable-interval (VI) schedule (30-45 seconds, VI35). Sucrose solution was also made available for each nose poke response initiated by subjects. If the number of recorded nose pokes and earned reinforcers equaled at least one by the end of the session, the subject was advanced to fixed-ratio (FR) 1 shaping sessions. If no responses were made to earn reinforcers, magazine training sessions were repeated for up to two additional consecutive days as necessary. Session advancement was determined on an individual basis.

#### 2.3.5 Fixed-ratio 1 shaping sessions

Subjects then underwent daily one-hour training sessions utilizing an FR 1 schedule of reinforcement to strengthen the association between nose poke behaviors and reinforcer availability. In these sessions, a reinforcer was made available for each nose poke response. Once subjects demonstrated consistent reward-related behavior by earning at least 20 reinforcers per session across two consecutive sessions, they were advanced individually to the progressive fixed-ratio (PFR) motivation test. If no responses were made across three consecutive sessions, subjects were individually returned to magazine training for one day before resuming FR 1 shaping.

#### 2.3.6 Progressive fixed-ratio breakpoint test

On the final day of the behavioral assay, subjects underwent a single session that utilized a PFR schedule of reinforcement, wherein reinforcers were administered with *n* + 1 responses where *n* represents the number of responses required to earn a reinforcer in the previous trial (i.e., 1 nose poke yields reinforcer availability on trial 1, 2 nose pokes yield reinforcer availability on trial 2, etc.). The breakpoint, or the point at which subjects stopped responding for a reinforcer, was measured as a proxy for motivation. A measure of average response effort was also derived by dividing the total number of nose pokes by the duration of time subjects engaged with the task. The session terminated after three hours or 20 minutes without an earned reinforcer, whichever occurred first.

### 2.4 Immunohistochemistry

#### 2.4.1 Tissue collection

Two hours after injections on the day after completion of the PFR breakpoint test, subjects were deeply anesthetized with isoflurane and sacrificed by rapid decapitation. Brains were removed and sagittally bisected for one hemisphere to be used in immunohistochemical analyses and the other to be used for lipidomic analyses with liquid chromatography-tandem mass spectrometry. The left hemisphere was postfixed in 4% paraformaldehyde (PFA) for 24 hours at room temperature, then an additional 24 hours at 4°C. Brains were agitated on a rocker for all steps to maximize tissue penetration. Following postfixation, brains were submerged in phosphate buffered saline (PBS) for three 20-minute washes, then transferred to 30% sucrose solution for 24 hours at 4°C. Prior to sectioning, brains were embedded with Tissue-Tek OCT compound, frozen with dry ice, and stored at -80°C. Sagittal sections 30 µm in thickness were cut with a Leica CM300 cryostat (Leica, Deerfield, IL). Sections for each subject were contained in sealed 12-well plates and stored in PBS at 4°C for immunolabeling. Three-four sections per subject were taken for each analysis.

#### 2.4.2 Tissue processing for histological analysis: neuronal dendrites (MAP2ab), microglia (Iba-1), astrocytes (GFAP), colocalization of IL-1β with astrocytes (GFAP/IL-1β)

All immunohistochemistry (IHC) wash and incubation procedures were performed at room temperature unless otherwise specified, and free-floating tissue was rocked at 27 rpm for all steps in 12-well plates fitted with 74 µm mesh inserts (Corning Life Sciences, Netwell Inserts, # 29442-132). All washes, as described, consist of three 5-minute rinses in phosphate buffer saline (PBS).

For MAP2ab and Iba-1 IHC, free-floating sections were first incubated in 0.5% H_2_O_2_ for 30 min, in 1% H_2_O_2_ for 60 min, and again in 0.5% H_2_O_2_ for 30 min, then washed, and followed by exposure to blocking buffer for 1 hour (PBS with 3% normal goat serum and 0.5% Triton X-100). Tissue was then incubated in primary antibodies, including microtubule-associated protein 2, ab (MAP2ab, mouse, Millipore, #MAB378; 1:500) for the detection of neuronal dendrites and ionized calcium-binding adapter molecule 1 (Iba-1, rabbit, Wako, #019-19741; 1:500) for the detection of microglia, mixed into blocking solution for 24 hours at 4°C. The primary antibodies were detected using secondary antibodies as follows: goat-anti-rabbit Alexa 594 (ThermoFisher, #A11012, red,1:500) and goat-anti-mouse Alexa 488 (ThermoFisher, #A21121, green, 1:500). The secondary antibodies were diluted in goat blocking buffer and applied to the sections for one hour. Cell nuclei were visualized with Hoechst 33342 (1:200, Molecular Probes, H3570, exposed for 3 minutes). Tissue sections were triple-rinsed in distilled water, mounted on Superfrost Plus glass microscopic slides (Fisher Scientific, #12-550-15), and coverslipped with antifade mounting medium (VectaShield, #H-1400).

For GFAP and IL-1β IHC, sections were washed and incubated in a permeability solution for 30 minutes [0.1% bovine serum albumin (BSA), 0.1% Triton X-100]. Samples were washed again and incubated in a blocking solution for 30 minutes (5% normal goat serum, 5% BSA, 0.1% Triton X-100). Tissue was then incubated in chicken anti-GFAP (1:1000; Thermo Fisher, #PA110004) and rabbit anti-IL-1β (1:250; Abcam, #AB9722) mixed into blocking solution for 24 hours at 4°C. Tissue was washed, covered, and incubated for one hour in goat anti-chicken Alexa Fluor 594 (1:500; Thermo Fisher, #A-11042) and goat anti-rabbit Alexa Fluor 488 (1:500; Thermo Fisher, #A-11034) diluted in blocking solution, washed, incubated for 3 minutes in Hoechst fluorescent stain (1:200 Hoechst:distilled water), then triple-rinsed in distilled water. Samples were then mounted on Superfrost Plus glass microscope slides and coverslipped with antifade mountant (Invitrogen ProLong Gold, #P36930).

#### 2.4.3 Confocal microscopy: mean fluorescence intensity, cell quantification, cell morphology, and colocalization analysis

Immunolabeled tissue was imaged at 20x or 63x using a Zeiss LSM800 T-PMT laser scanning confocal microscope and ZEN 2018 Blue Edition software (Carl Zeiss, Inc., Thornwood, NY). The collection, processing and analyses of all images were conducted by experimenters blind to genotype and drug conditions. Images were acquired by using identical parameters for all groups (i.e., identical objective, zoom, laser intensity, gain, offset, and scan speed) optimized for control tissues. For both brain regions (i.e., nucleus accumbens and infralimbic cortex), images were sampled from 3-4 sagittal sections, spaced 300 μm apart, per animal.

For MAP2ab^+^ immunoreactive neuronal dendrites, one image per brain region was taken per section, and the entire image (20x objective, 0.3 mm^2^) was used as region of interest and processed using ImageJ (70) to quantify the intensity of staining per pixel in each image. Mean fluorescent intensity (MFI) was determined with ImageJ without digital manipulation. Data represent individual subject data (MAP2ab MFI) averaged across all images taken per brain region.

For microglia activity, one image per brain region was taken per section and Iba-1^+^ microglial cell bodies containing Hoechst-stained nuclei were counted by two experimenters blinded to treatment groups (20x objective, 0.3 mm^2^). Interrater reliability was assessed using Cronbach’s alpha (nucleus accumbens α = .798; infralimbic cortex α = .814), and data from both raters were averaged to represent microglia counts. Data represent individual subject data (Iba-1^+^ microglia counts) averaged across all images taken per brain region. Furthermore, microglia morphology was assessed for the nucleus accumbens via Sholl analysis following published procedures (63, 71, 72). In brief, z-stack images were collected at 63x magnification of immunodetected Iba-1^+^ microglia stained with Hoechst (*n* = 4 mice per group/3-4 sections each). Orthogonal projection images were generated from the slide z-stack images, resulting in one z-plane, using ZEN 2018 Blue Edition software, and exported to Fiji build of ImageJ. Per animal a total of 5-6 microglia were individually isolated for analysis by random selection (72). The soma size was measured in Fiji using the freehand selection tool. The images were cleaned, and the background noise was removed using the despeckle tool. The processed images were overlayed with the original image, and the tracing tool was used to select the microglia of interest, and the background was cleared. The images were converted to binary images, and the line segment tool was used to draw a line from the center of each soma to the tip of its longest process, providing the maximum branch length (μm). The Sholl analysis plugin software was used to assess additional measures, with the first shell set at 10 μm and subsequent shells set at 2 μm sizes, to determine intersections at each Sholl radius. This provided the critical radius (radius value with the highest number of intersections), the process maximum (the highest number of intersections regardless of radius value), the number of primary processes (intersections at the first Sholl radius), and the process total (total number of intersections). Individual microglia were treated as individual data points.

For GFAP^+^ astrocytes and colocalization with IL-1β, three z-stack images per brain region were taken per section (20x objective, 0.3 mm^2^), and ImageJ software was used for astrocyte quantification and analyses of colocalization with IL-1β. GFAP^+^ astrocyte cell bodies containing Hoechst-stained nuclei were manually quantified by two experimenters blinded to treatment groups. Interrater reliability was assessed using Cronbach’s alpha (α = 0.993) (nucleus accumbens α = .994; infralimbic cortex α = .992), and data from both raters were averaged to represent astrocyte counts. Data represent individual subject data (GFAP^+^ astrocyte counts) averaged across all images taken per brain region. For GFAP^+^ colocalization with IL-1β, the JACoP plugin was used for colocalization measures (73). Herein, absolute intensity thresholds were defined manually for both channels to control for any background fluorescence. Voxels that exceeded thresholds across both channels were considered colocalized, and the corresponding Pearson’s correlation coefficient (PCC) was recorded. Generated cytofluorograms were also reviewed to ensure accurate colocalization thresholds. Data represent individual subject data (GFAP/IL-1β colocalization) averaged across all images taken per brain region. Note, that we lost the brain sections of one vehicle-treated Tat(−) mouse due to a processing error during IHC labeling, and thus, only 8 individual subject data points are shown for GFAP^+^ astrocyte counts and GFAP/IL-1β colocalization.

### 2.5 Liquid chromatography-tandem mass spectrometry

#### 2.5.1 Metabolo-lipidomic sample preparation

Whole tissue striatal and hippocampal samples dissected from the hemisphere that were not used for IHC were prepared as previously described (74). All standards and internal standards used for LC-MS/MS analysis (referred to collectively as lipidomic analyses elsewhere) of arachidonic acid, docosahexaenoic acid, and linoleic acid-derived lipid mediators were purchased from Cayman Chemical (Ann Arbor, Michigan, USA). All high-performance liquid chromatography (HPLC) solvents and extraction solvents were HPLC grade or better.

Tissue samples were massed into pre-chilled (-20°C) Qiagen homogenizer tubes containing a 5mm stainless steel homogenizing bead (Qiagen, Germantown, MD, USA). An aliquot of 500 µL pre-chilled methanol was added to each tube before homogenizing at 50 Hz for 2 minutes. Samples were then centrifuged at 14,000 rpm and 4°C for ten minutes. The supernatant was extracted into a 1.5 mL centrifuge tube and spiked with 10 µL of the internal standard solution (10 pg/µL each of 5(S)-HETE-d8, 8-iso-PGF2a-d4, 9(S)-HODE-d4, LTB4-d4, LTD4-d5, LTE4-d5, PGE2-d4, PGF2a-d9 and RvD2-d5 in ethanol), followed by vortexing. The sample was then dried in a vacuum centrifuge at 55°C until dry. The sample was then immediately reconstituted in 1.0 mL of 90:10 water:methanol before purification by solid phase extraction (SPE).

Lipid mediators were isolated using Strata-X 33 µm 30 mg/1 mL SPE columns (Phenomenex, Torrance, CA) on a Biotage positive pressure SPE manifold (Biotage, Charlotte, NC). SPE columns were pre-washed with 2 mL of methanol (MeOH) followed by 2 mL of H_2_O. After applying the entire 1 mL of reconstituted sample, the columns were washed with 1 mL of 10% MeOH. The lipid mediators were then eluted sequentially with 1 mL of methyl formate followed by 1 mL of MeOH directly into a reduced surface activity/maximum recovery glass autosampler vial (MicroSolv Technology Corp. Leland, NC), drying after each solvent elution with a steady stream of nitrogen directly on the SPE manifold. The sample was then immediately reconstituted with 20 µL of ethanol and analyzed immediately or stored at –70°C until analysis for no more than 1 week.

#### 2.5.2 Liquid chromatography-mass spectrometry

Quantitation of lipid mediators was performed using 2-dimensional reverse phase HPLC tandem mass spectrometry (LC-MS/MS). The HPLC system consisted of an Agilent 1260 autosampler (Agilent Technologies, Santa Clara, CA), an Agilent 1260 binary loading pump (pump 1), an Agilent 1260 binary analytical pump (pump 2), and a 6-port switching valve. Pump 1 buffers consisted of 0.1% formic acid in water (solvent A) and 9:1 v:v acetonitrile:water with 0.1% formic acid (solvent B). Pump 2 buffers consisted of 0.01% formic acid in water (solvent C) and 1:1 v:v acetonitrile:isopropanol (solvent D).

Five µL of extracted sample was injected onto an Agilent SB-C18 2.1 X 5 mm 1.8 µm trapping column using pump 1 at 2 mL/minute for 0.5 minutes with a solvent composition of 97% solvent A: 3% solvent B. At 0.51 minutes, the switching valve changed the flow to the trapping column from pump 1 to pump 2. The flow was reversed and the trapped lipid mediators were eluted onto an Agilent Eclipse Plus C-18 2.1 X 150 mm 1.8 µm analytical column using the following gradient at a flow rate of 0.3 mL/minute: hold at 75% solvent A:25% solvent D from 0-0.5 minutes, then a linear gradient from 25-75% D over 20 minutes followed by an increase from 75-100% D from 20-21 minutes, then holding at 100% D for 2 minutes. During the analytical gradient, pump 1 washed the injection loop with 100% B for 22.5 minutes at 0.2 mL/minute. Both the trapping column and the analytical column were re-equilibrated at starting conditions for 5 minutes before the next injection.

Mass spectrometric analysis was performed on an Agilent 6490 triple quadrupole mass spectrometer in negative ionization mode. The drying gas was 250°C at a 15 mL/minute flow rate. The sheath gas was 350°C at 12 mL/minute. The nebulizer pressure was 35 psi. The capillary voltage was 3500 V. Data for lipid mediators was acquired in dynamic MRM mode using experimentally optimized collision energies obtained by flow injection analysis of authentic standards. Calibration standards for each lipid mediator were analyzed over a range of concentrations from 0.25 – 250 pg on column. Calibration curves for each lipid mediator were constructed using Agilent Masshunter Quantitative Analysis software. Tissue samples were quantitated using the calibration curves to obtain the on-column concentration, followed by multiplication of the results by the appropriate dilution factor to obtain the concentration in pg/mL.

### 2.6 Statistical analyses

All statistical analyses were performed using SPSS (IBM SPSS Statistics, Version 28, Chicago, IL, USA) and represented visually using GraphPad Prism (GraphPad Software, Inc., Version 9, San Diego, CA, USA) and OriginPro (OriginLab Corporation, Version 2022b, Northampton, MA, USA). Biorender was also utilized to create the experimental schematic depicted in **Figure 1**. Data are reported as mean ± standard error. Two-way analyses of variance (ANOVAs) with follow-up Tukey’s *post hoc* tests when appropriate were conducted with genotype [2 levels: Tat(−), Tat(+)] and drug (2 levels: vehicle, MJN110) as between-subjects factors for all but three measures (exceptions detailed as follows). For assessments of body mass, three-way mixed ANOVAs were conducted with time (14 levels: 14 days) as a within-subjects factor and genotype and drug as between-subjects factors. This was then followed up by two-way ANOVAs with genotype and drug as between-subjects factors for each day and Tukey’s *post hoc* tests when appropriate. For assessments of PFR breakpoint and number of days to acquisition, Shapiro Wilk tests demonstrated residuals failed to meet normality assumptions; as such, Mann-Whitney U nonparametric tests were run to assess the effects of genotype and drug. Pearson correlation coefficients were calculated for three behavioral measures and all striatal-related CNS measures, except for Iba-1^+^ microglial morphology due to the chosen smaller sample size, and potential relationships between variables were explored. Data were subdivided by genotype and drug groups to obtain the correlation matrices. Pearson’s correlation coefficients were calculated for each pair of continuous variables for each group. This allowed the assessment of the strength and direction of the linear relationship between variables within each group separately. An alpha of *p* ≤ 0.05 was considered significant for all analyses.

## 3 Results

### 3.1 Body mass was not affected by chronic MJN110 treatment

**Figure 2:**
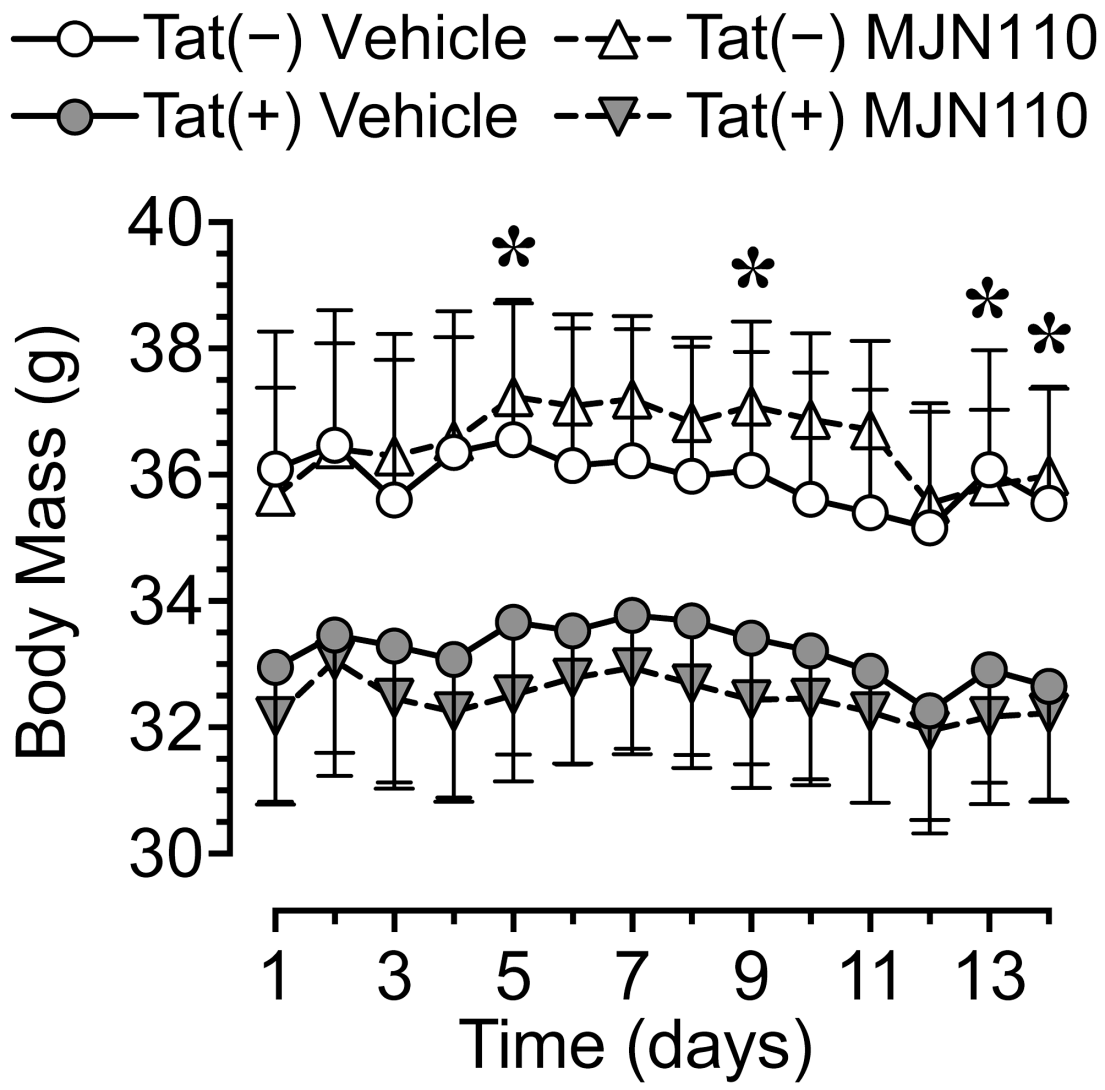
Body mass data for the two weeks of MJN110/vehicle injections prior to the start of behavioral assessment. Tat expression showed a trend for decreased body mass (g) over the two-week period, with lower body mass for Tat(+) mice compared to Tat(−) mice on days 5, 9, 13, and 14. Follow-up Tukey’s *post hoc* test demonstrated no significant group differences. No effects were noted for MJN110 treatment. All data are expressed as mean ± the standard error of the mean (SEM). Statistical significance was assessed by ANOVAs followed by Tukey’s *post hoc* tests when appropriate; *p < 0.05 main effect of genotype.

Body mass (g) was taken daily from the start of drug injections until the end of the study. Body mass data are reported for the first two weeks of drug injections prior to the start of behavioral assessment. No significant effects were noted, with only a trend toward a significant genotype effect [*F*(1, 30) = 3.7, *p* = 0.063; **Figure 2**]. Separate two-way ANOVAs for each day revealed Tat expression significantly decreasing body mass for day 5 [*F*(1, 30) = 4.3, *p* = 0.047], day 9 [*F*(1, 30) = 4.7, *p* = 0.038], day 13 [*F*(1, 30) = 4.5, *p* = 0.042], and day 14 [*F*(1, 30) = 4.2, *p* = 0.050]. Follow-up Tukey’s *post hoc* tests demonstrated no significant group differences.

### 3.2 No behavioral alterations were observed following Tat induction or MJN110 treatment

**Figure 3:**
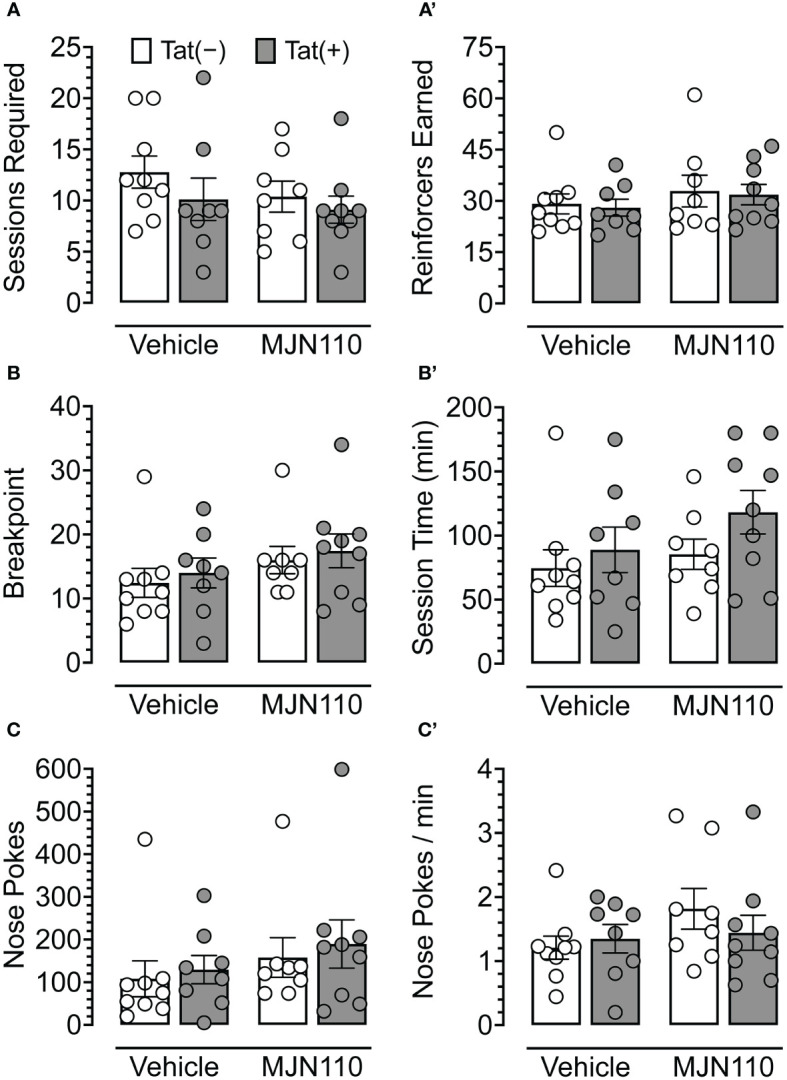
Behavioral data for fixed-ratio (FR) 1 shaping sessions and progressive fixed-ratio (PFR) breakpoint test. For FR 1 shaping **(A, A’)**, no significant differences were observed between genotype or drug groups for the number of sessions required to advance to the PFR breakpoint test **(A)** or the number of reinforcers earned in sessions wherein criteria for advancement were met **(A’)**. In the PFR breakpoint test **(B-C’)**, no significant differences were observed for breakpoint **(B)**, session length **(B’)**, total number of nose poke behaviors **(C)**, or nose pokes per minute **(C’)**. All data are expressed as mean ± the standard error of the mean (SEM). Statistical significance was assessed by ANOVAs or Mann-Whitney U nonparametric tests. Individual subject data are represented by open circles.

No baseline performance differences were observed between Tat(−) and Tat(+) subjects across drug groups during FR 1 shaping trials, either in the number of sessions required to learn the task (**Figure 3A**) or the number of reinforcers earned during sessions wherein criteria for advancement to the PFR breakpoint test were met (**Figure 3A’**). Additionally, no significant differences were observed between Tat(−) and Tat(+) subjects across drug groups during the PFR breakpoint test in measures of breakpoint (**Figure 3B**), total session time (**Figure 3B’**), number of nose pokes (**Figure 3C**), or nose pokes per minute (**Figure 3C’**). These results indicate that Tat and 1 mg/kg MJN110 demonstrate no significant effects on either reward-related task learning or motivation to exert additional effort to receive a salient reinforcer.

### 3.3 MJN110 reversed Tat-induced dendritic injury, but only minor effects were noted on microglia

#### 3.3.1 Neuronal dendritic density

**Figure 4:**
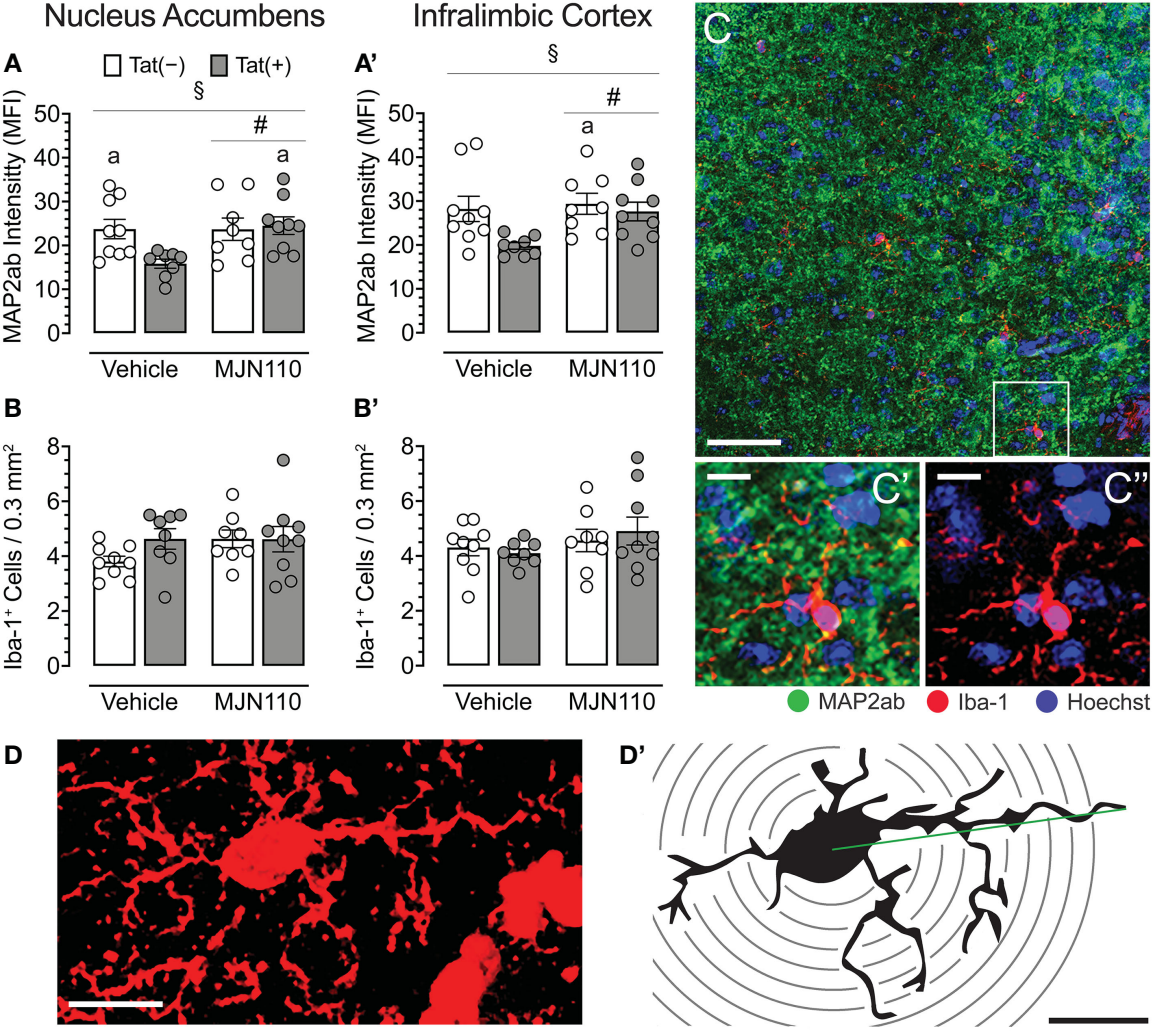
Neuronal dendritic intensity and microglia density/morphology in the nucleus accumbens and infralimbic cortex. **(A)** In the nucleus accumbens, MJN110 increased neuronal dendritic intensity based on Tat expression, with decreased MAP2ab intensity in vehicle-treated Tat(+) mice compared to MJN110-treated Tat(+) mice and vehicle-exposed Tat(−) mice. **(A’)** In the infralimbic cortex, the same main effect for drug and drug x genotype interaction was noted, with decreased MAP2ab intensity in vehicle-treated Tat(+) mice compared to MJN110-treated Tat(−) mice. **(B, B’)** Microglia density was not affected by genotype or drug in the nucleus accumbens or infralimbic cortex. **(C)** Brain section from the nucleus accumbens of a vehicle-treated Tat(+) mouse taken at 20x (0.3 mm^2^, Scale bar = 50 µm) and at a higher magnification of MAP2ab intensity and/or microglia Iba-1 (**C’** and **C’’**, Scale bars = 10 μm). **(D)** Example of an orthogonal projection Iba-1-stained image from the nucleus accumbens used for Sholl analysis (63x, Scale bar = 10 μm, image processed with ImageJ). **(D’)** Example of a single microglial cell with concentric Sholl radii (black circles) superimposed on the image (Scale bar = 10 μm). All data are expressed as mean ± the standard error of the mean (SEM). Statistical significance was assessed by ANOVAs followed by Tukey’s *post hoc* tests when appropriate; ^#^ *p* < 0.05 main effect of drug, ^§^ *p* < 0.05 genotype x drug interaction, ^a^ *p* < 0.05 vs. vehicle-treated Tat(+) mice. Individual subject data (averaged across 3-4 images per region) represented by open circles.

MJN110 significantly increased neuronal dendritic density in the nucleus accumbens [*F*(1, 29) = 4.4, *p* = 0.044; **Figure 4A**], which was significantly altered by genotype [*F*(1, 29) = 4.5, *p* = 0.042]. A follow-up Tukey’s *post hoc* test revealed vehicle-treated Tat(+) mice showing lower neuronal dendritic density compared to vehicle-treated Tat(−) mice (*p* = 0.050) and MJN110-treated Tat(+) mice (*p* = 0.027). Within the infralimbic cortex, significant effects of both Tat and MJN110 were observed [*F*(1, 29) = 5.2, *p* = 0.030 and *F*(1, 29) = 4.0, *p* = 0.054, respectively; **Figure 4A’**]. A follow-up Tukey’s *post hoc* test revealed vehicle-treated Tat(+) mice showing lower neuronal dendritic density compared to MJN110-treated Tat(−) mice (*p* = 0.030). No other group comparisons were significant, with only trending towards significance for vehicle-treated Tat(+) mice compared to MJN110-treated Tat(+) mice (*p* = 0.057).

#### 3.3.2 Microglia density and morphology

For both brain regions, the nucleus accumbens and the infralimbic cortex, Tat and MJN110 had no significant effects on microglia density (**Figure 4B, B’, C, C’, C’’**). Sholl analyses were conducted in the nucleus accumbens to assess microglia morphology (*n* = 4 mice per group with 3-4 sections/5-6 microglia, **Figures 4D, D’** and **Table 1**). Tat expression significantly increased the soma area (**Table 1**), which is associated with amoeboid morphology (71) and has been reported previously for Tat(+) male mice (63). Tukey’s *post hoc* test revealed no significant differences between groups. Additionally, MJN110 increased the number of primary processes (intersections at the first Sholl radius), whereas no other measure was affected by the drug, and Tukey’s *post hoc* test revealed no significant differences between groups. Overall, microglia morphology appears to be altered by Tat and MJN110 treatment only to a minimal extent.

**Table 1:** Effect of Tat and MJN110 on microglia morphology in the nucleus accumbens of Tat transgenic mice.

Measure	Genotype	Repeated vehicle	Repeated MJN110	Genotype effect		Drug effect		Genotype x drug	
		mean ± SEM	mean ± SEM	*F*_1,80_	*p*	*F*_1,80_	*p*	*F*_1,80_	*p*
Soma area (µm^2^)	Tat(–)	30.85 ± 1.16	30.87 ± 1.02	3.92	**0.05**	0.86	0.35	0.90	0.34
	Tat(+)	33.82 ± 0.99	31.92 ± 0.85						
Maximum branch length (µm)	Tat(–)	29.24 ± 1.26	30.06 ± 1.33	0.18	0.66	0.17	0.68	0.04	0.84
	Tat(+)	30.08 ± 1.32	30.36 ± 1.39						
Critical radius (µm)	Tat(–)	12.76 ± 0.56	12.60 ± 0.63	1.94	0.16	0.003	0.95	0.02	0.86
	Tat(+)	13.64 ± 0.81	13.71 ± 0.78						
Number of primary process	Tat(–)	4.95 ± 0.34	5.90 ± 0.53	0.13	0.71	4.64	**0.03**	0.004	0.95
	Tat(+)	5.09 ± 0.53	6.10 ± 0.35						
Process maximum	Tat(–)	6.71 ± 0.44	7.65 ± 0.47	0.04	0.82	2.88	0.09	0.08	0.77
	Tat(+)	6.95 ± 0.44	7.62 ± 0.34						
Process total	Tat(–)	38.19 ± 3.84	38.95 ± 2.22	0.51	0.47	0.48	0.48	0.20	0.65
	Tat(+)	39.00 ± 3.23	42.57 ± 2.77						

Sholl analysis of microglia morphology in the nucleus accumbens of repeated (2-week) vehicle- or MJN110-treated Tat(–) and Tat(+) female mice. Data are expressed as the mean ± SEM. The parameters measured by Sholl analysis are indicated in parentheses in the first column. Two-way ANOVAs for each measurement were conducted with genotype and drug as between-subject factors. F values and p values are presented from ANOVA results. Bolded values denote significant differences at *p* < 0.05; mean ± SEM, *n* = 4 mice per group with 3-4 sections/5-6 microglia.

### 3.4 MJN110 reversed some Tat-induced alterations to astrocyte density and IL-1β recruitment

#### 3.4.1 Astrocyte density

**Figure 5:**
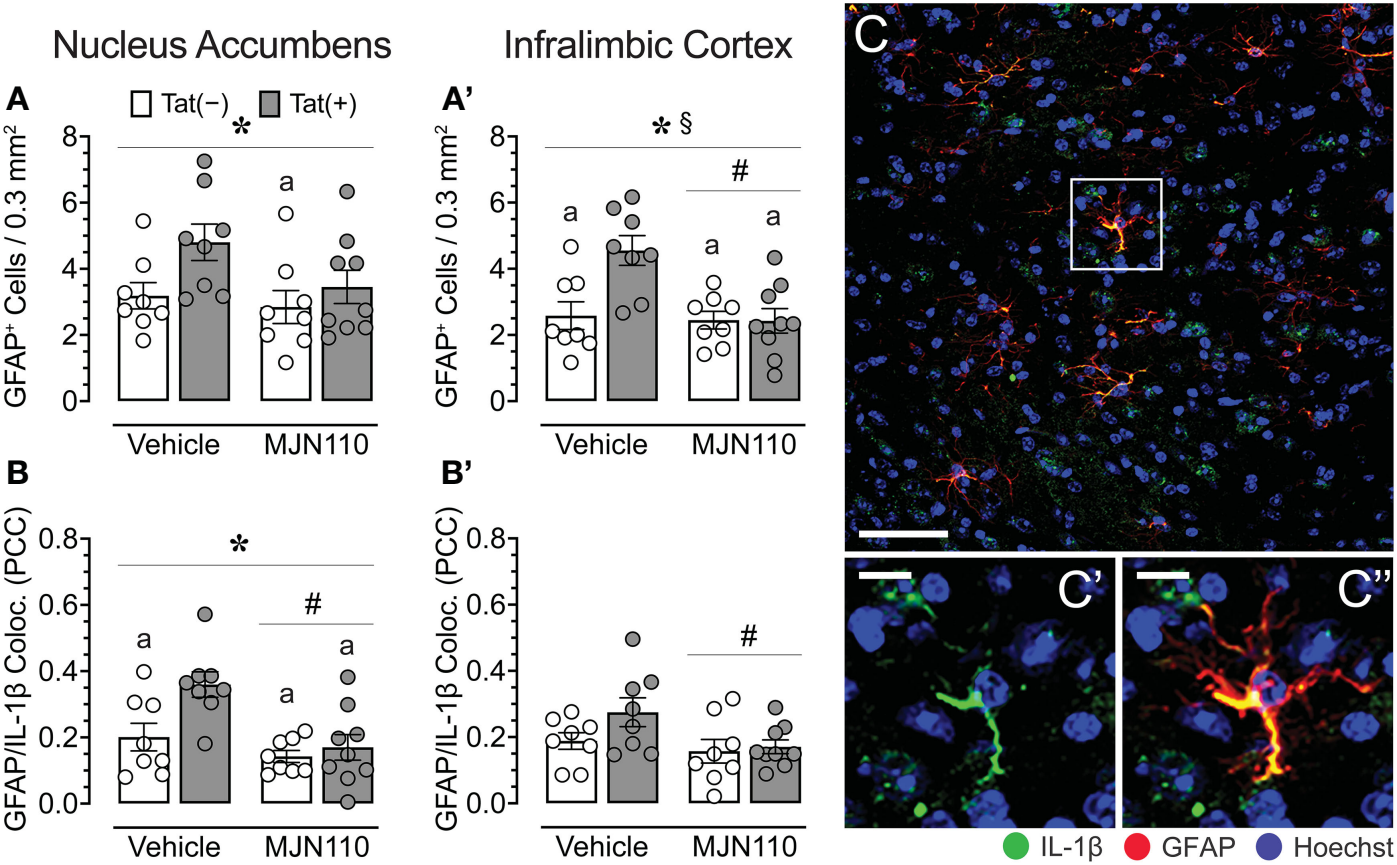
Astrocyte density and astrocytic IL-1β colocalization in the nucleus accumbens and infralimbic cortex. **(A)** Tat increased astrocyte density in the nucleus accumbens, with increased GFPA^+^ counts for vehicle-treated Tat(+) mice compared to MJN110-treated Tat(−) mice. **(A’)** In the infralimbic cortex, MJN110 downregulated Tat-driven increases in astrocyte density based on Tat expression, with increased GFPA^+^ counts for vehicle-exposed Tat(+) mice compared to all other groups. **(B)** Colocalization of IL-1β with GFAP^+^ cells in the nucleus accumbens was significantly affected by Tat and MJN110, with vehicle-exposed Tat(+) mice showing higher colocalization of IL-1β with GFAP^+^ cells compared to all other groups. **(B’)** In the infralimbic cortex, colocalization of IL-1β with GFAP^+^ cells was downregulated by MJN110. **(C)** Brain section of GFAP^+^ astrocytes with colocalization of IL-1β from the nucleus accumbens of a vehicle-treated Tat(+) mouse taken at 20x (0.3 mm^2^, Scale bar = 50 µm) and at a higher magnification (**C’** and **C’’**, Scale bars = 10 μm). All data are expressed as mean ± the standard error of the mean (SEM). Statistical significance was assessed by ANOVAs followed by Tukey’s *post hoc* tests when appropriate; *p < 0.05 main effect of genotype, ^#^ *p* < 0.01 main effect of drug, ^§^ *p* < 0.05 genotype x drug interaction, ^a^ *p* < 0.05 vs. vehicle-treated Tat(+) mice. Individual subject data (averaged across 3-4 images per region) are represented by open circles. Note that the brain sections of one vehicle-treated Tat(−) mouse were lost due to a processing error during IHC labeling and thus, only 8 individual subject data points are shown for the vehicle-treated Tat(−) mouse group. PCC, Pearson’s colocalization coefficient.

Tat significantly increased astrocyte density in the nucleus accumbens [*F*(1, 29) = 5.1, *p* = 0.032; **Figure 5A**]. Subjects treated with MJN110 trended towards a significant decrease in this region [*F*(1, 29) = 3.0, *p* = 0.096]. A follow-up Tukey’s *post hoc* test revealed vehicle-treated Tat(+) mice showing higher astrocyte density compared to MJN110-treated Tat(−) mice (*p* = 0.045). In the infralimbic cortex (**Figure 5A’**), Tat significantly increased the number of GFAP-positive astrocytes [*F*(1, 29) = 6.5, *p* = 0.017], whereas MJN110 significantly decreased this measure [*F*(1, 29) = 8.7, *p* = 0.006]. Importantly, a significant interaction was observed between genotype and drug [*F*(1, 29) = 6.8, *p* = 0.015], in which vehicle-treated Tat(+) subjects showed higher astrocyte density compared to all other groups [vehicle-treated Tat(−) mice, *p* = 0.006; MJN110-treated Tat(−) mice, *p* = 0.003; MJN110-treated Tat(+) mice, *p* = 0.002].

#### 3.4.2 Astrocytic IL-1β colocalization

Within the nucleus accumbens, significant effects of both, Tat and MJN110 were observed [*F*(1, 29) = 6.7, *p* = 0.015 and *F*(1, 29) = 12.0, *p* = 0.002, respectively], as well as a trend towards a significant interaction [*F*(1, 29) = 3.3, *p* = 0.078; **Figure 5B**). Notably, a follow-up Tukey’s *post hoc* test revealed that vehicle-treated Tat(+) subjects showed higher colocalization of IL-1β with GFAP compared to all other groups [vehicle-treated Tat(−) mice, *p* = 0.022; MJN110-treated Tat(−) mice, *p* = 0.001; MJN110-treated Tat(+) mice, *p* = 0.004]. While in the infralimbic cortex (**Figure 5B’**), no main effect of Tat was observed for colocalization of IL-1β with GFAP, MJN110 significantly decreased colocalization across genotypes [*F*(1, 29) = 4.5, *p* = 0.042]. A follow-up Tukey’s *post hoc* test demonstrated no significant group differences. **Figures 5C-C''** shows a representative image from the nucleus accumbens of colocalization of IL-1ß with GFAP^+^ astrocytes.

### 3.5 Contrasting effects of Tat and MJN110 on proinflammatory lipid mediator expression

**Figure 6:**
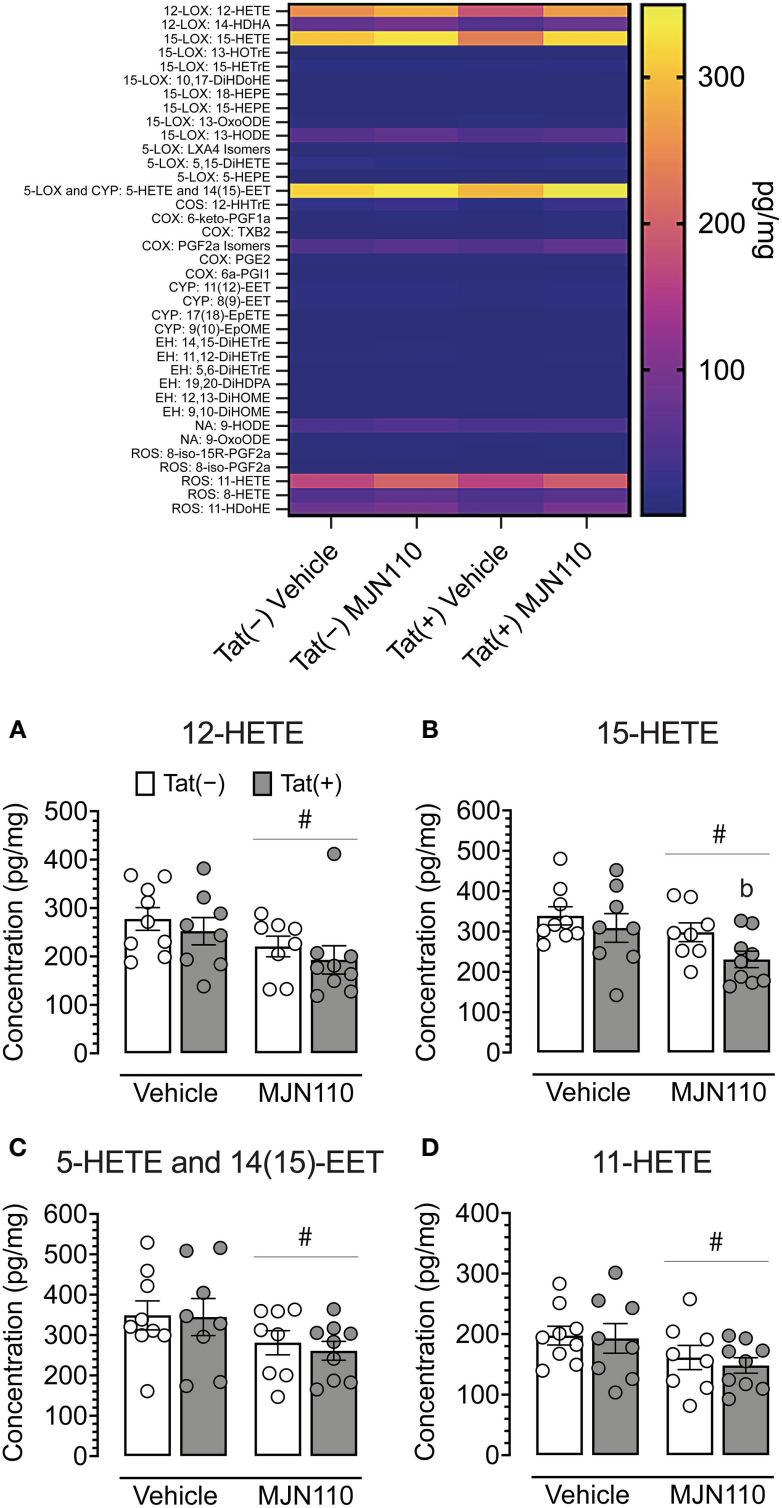
Quantification of striatal lipid mediators. Heatmap of all lipid mediators assessed (top). Summary data from significant findings **(A–D)**. MJN110 treatment significantly decreased striatal 12-HETE **(A)**, 15-HETE **(B)**, 5-HETE/14(15)-EET **(C)**, and 11-HETE **(D)**. All data are expressed as mean ± the standard error of the mean (SEM). Statistical significance was assessed by ANOVAs followed by Tukey’s *post hoc* tests when appropriate; ^#^ *p* < 0.05 main effect of drug, ^b^ *p* = 0.023 vs. vehicle-treated Tat(−) mice. Individual subject data are represented by open circles.

Due to the more prominent astrocytic IL-1β colocalization observed in the nucleus accumbens relative to the infralimbic cortex and our focus of interest on associated inflammatory lipid mediators, lipidomic analyses with LC-MS/MS centered primarily on further characterizing these profiles within the striatum. These analyses revealed a significant main effect of MJN110 treatment in reducing striatal 12-HETE [*F*(1, 30) = 5.0, *p* = 0.033; **Figure 6A**], 15-HETE [*F*(1, 30) = 5.3, *p* = 0.029; **Figure 6B**], 5-HETE/14(15)-epoxyeicosatrienoic acid [EET; *F*(1, 30) = 4.9, *p* = 0.035; **Figure 6C**], and 11-HETE [*F*(1, 30) = 4.7, *p* = 0.038; **Figure 6D**). Follow-up Tukey’s *post hoc* tests revealed decreased 15-HETE striatal levels for MJN110-treated Tat(+) mice compared to vehicle Tat(−) mice (*p* = 0.023). Additional alterations were observed in the hippocampus (see **Supplementary Figure S1**). These analyses revealed a significant genotype x drug interaction for 12-HETE [*F*(1, 30) = 4.6, *p* = 0.040], with follow-up Tukey’s *post hoc* revealing no significant group differences. Further, significant main effects of MJN110 treatment in reducing hippocampal 15-HETE [*F*(1, 30) = 4.5, *p* = 0.042] and 5-HETE/14(15)-epoxyeicosatrienoic acid [EET; *F*(1, 30) = 6.7, *p* = 0.015] were noted. Further, Tat expression increased 11-HETE [*F*(1, 30) = 4.4, *p* = 0.046] that was altered by drug [genotype x drug interaction, *F*(1, 30) = 4.1, *p* = 0.051]. Follow-up Tukey’s *post hoc* tests revealed increased 11-HETE hippocampal levels for vehicle-treated Tat(+) mice compared to vehicle Tat(−) mice (*p* = 0.032).

### 3.6 Subgroup-specific relationships between measures

**Figure 7:**
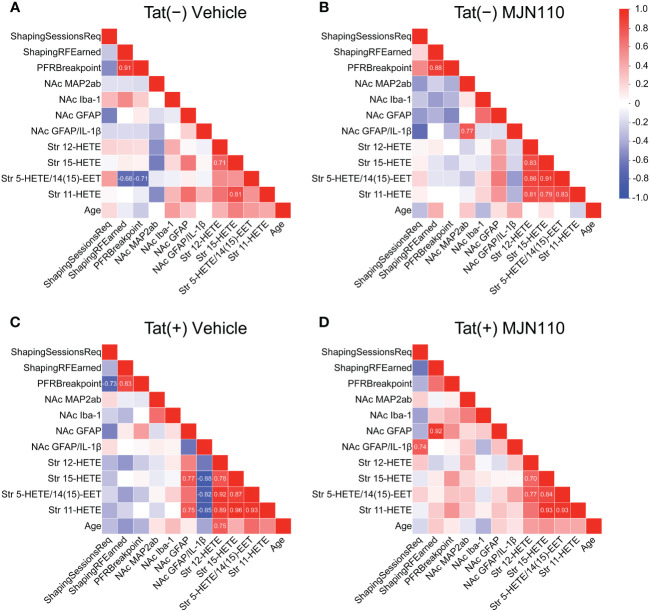
Correlation matrices of quantified variables across groups. Association patterns between behavior, nucleus accumbens MAP2ab+ dendritic intensity, microglia density, astrocyte density, astrocytic IL-1β colocalization, and striatal inflammatory lipid mediator expression differ between subgroups. Among other distinctions, predictors of PFR breakpoint varied between genotype and treatment groups. Additionally, vehicle-treated Tat(+) subjects **(C)** show stronger associations between astrocyte density and proinflammatory lipid mediator expression relative to the other groups, vehicle-treated Tat(−) mice **(A)**, MJN110-treated Tat(−) subjects **(B)**, and MJN110-treated Tat(+) mice **(D)**. Pearson’s correlation coefficients (*r*) are indicated for significant values at *p* ≤ 0.05. NAc, nucleus accumbens.

Associations between CNS and behavioral measures differed among subgroups and were only found in vehicle-treated Tat(−) mice and MJN110-treated Tat(+) subjects (**Figure 7**). In vehicle-treated Tat(−) subjects, low striatal 5-HETE/14(15)-EET was strongly associated with higher breakpoints and a larger number of reinforcers earned during FR1 shaping sessions (**Figure 7A**). In contrast, for MJN110-treated Tat(+) subjects, increased astrocyte density and higher astrocytic IL-1β colocalization in the nucleus accumbens was strongly associated with a larger number of reinforcers earned during FR1 shaping sessions and a greater number of shaping sessions required to advance to the PFR test, respectively (**Figure 7D**).

Behavioral predictors of PFR breakpoint also varied between groups. For all groups, except MJN110-treated Tat(+) mice, a larger number of reinforcers earned during FR1 shaping sessions was strongly associated with higher PFR breakpoints (**Figures 7A-C**). Further, in vehicle-treated Tat(+) subjects, a lower number of required FR1 shaping sessions was strongly associated with higher PFR breakpoints (**Figure 7C**).

Associations between CNS measures demonstrated that the four striatal HETE mediators showed high positive correlations within each other for all groups, except for the vehicle-treated Tat(−) mice (**Figures 7B-D**). In vehicle-treated Tat(−) mice, only higher striatal 15-HETE levels were associated with higher 11-HETE and 12-HETE levels (**Figure 7A**). Interestingly, in vehicle-treated Tat(+) subjects, higher striatal 15-HETE and 11-HETE levels were strongly associated with increased astrocyte density in the nucleus accumbens, whereas higher striatal HETEs were also associated with less IL-1β colocalization with GFAP (**Figure 7C**).

## 4 Discussion

While no behavioral effects were observed, the present study demonstrated that in the infralimbic cortex and nucleus accumbens, MJN110 successfully reduced Tat-induced dendritic injury and Tat-induced astrocyte-related neuroinflammation, as shown by decreasing Tat-induced upregulated astrocyte density or astrocytic IL-1β colocalization. Notably, Tat induced region-specific inflammatory effects such that astrocyte recruitment was increased in the infralimbic cortex, whereas instead, astrocytic IL-1β was increased in the nucleus accumbens. No effects were noted for Iba-1^+^ microglia density in either brain region, and only minor effects were noted for microglia morphology assessed in the nucleus accumbens. Further, selected HETE-related inflammatory lipid mediators in the striatum were downregulated by chronic MJN110 treatment.

The absence of behavioral differences, while contrary to hypothesized outcomes, may indeed be of some significance insofar as these results demonstrate MJN110 lacks cannabimimetic effects. Tetrahydrocannabinol (THC), a major CB_1_R agonist component of cannabis, has been shown to increase dopamine signaling in the nucleus accumbens as well as the ventral tegmental area (75–77), with both regions being critical components of the mesocorticolimbic system involved in reward-related behaviors and salience processing. These THC-mediated dopamine alterations underlie commonly observed increases in food intake, often referred to as the “munchies” (78). While other studies of chronic THC use have contrastingly found decreases in dopamine synthesis and activity (79), it is important to note these results are largely driven by cannabis-dependent individuals with high-severity use (80, 81). The limited existing data available for MJN110 effects on motivated behavior demonstrate that acute treatment increases reward-directed response; however, this effect was only observed at doses five- and tenfold higher than the 1 mg/kg dose used in the present study (61).

The lack of behavioral alterations across groups may also be explained by compensatory mechanisms such as homeostatic scaling (82). Specifically, excitatory synapse loss and increased inhibitory tone have previously been observed, both of which function to protect neurons from increased N-methyl-D-aspartate (NMDA) receptor activity in the presence of Tat-induced excitotoxicity (26, 83). Previous work has also found immune tolerance driven by Tat when assessing microglia activity [65]. As these compensatory changes in excitability and immune response are evident in the context of chronic rather than acute Tat exposure (63, 84), it could be speculated that more robust behavioral alterations may have been observed earlier after Tat induction than the time period presently investigated. Immune tolerance may also contribute to the lack of Tat-induced effects on lipid metabolite expression observed presently; while Tat initially induces inflammation, potentially contributing to dendritic injury seen in vehicle-exposed Tat(+) mice, the immune system becomes desensitized to its effects after about three months of exposure (63). Reduction in lipid metabolite expression following MJN110 treatment may suggest the potential to modulate immune responses in the chronic phase of HIV-1-associated neuroinflammation wherein observable inflammatory insult is diminished, but immune priming remains present (85). While no differences were observed between Tat(+) and Tat(−) subjects, the lipid metabolites assessed may not be as sensitive to Tat exposure in chronic conditions. MJN110 may exert effects through other mechanisms (e.g., modulation of immune cell activity or regulation of downstream signaling pathways associated with neuroinflammation).

Another plausible alternative explanation for behavioral similarity across groups is the growing body of research demonstrating biological sex-specific effects of HIV-1 and constituent viral proteins, including Tat, on both peripheral and CNS outcomes (86). Homeostatic scaling through upregulation of inhibitory activity by γ-aminobutyric acid (GABA)ergic neurons in the hippocampus (26, 83) and the prefrontal cortex have been established in the context of Tat expression, with the latter particularly in females (87). In accordance with these neurophysiological data, studies focused on neurocognitive outcomes of infection have shown worsened behavioral profiles among infected females in domains of spatial memory, learning, and motor skills (88, 89). Data have also shown female-specific hypersensitivity to pain (90), slower recovery from nerve injury, and heightened inflammatory responses to local injury (91). As sex-specific findings regarding executive function have historically been inconsistent (92, 93) and relatively little has been established for motivational deficits, the addition of male subjects in the present work would have provided valuable insight into potential sex differences in these domains. In addition to between-sex variation, there may be within-sex variation due to estrous cycle. Previous studies employing similar models have demonstrated that HIV-1 affects motivation, including reduced response vigor and increased reward threshold (28, 94); however, female subjects were not included in those studies. Prior work has also shown decreased motivation for food rewards (92, 95), even under food-restricted conditions when in estrus, during which time attention is diverted preferentially to sexual motivation (95). It has been posited that this decrease in consumption during estrus occurs due to reductions in positive-feedback signals elicited by natural reinforcers (96), potentially a result of increased striatal dopamine turnover and decreased dopamine concentrations during this phase of the estrous cycle (97). Tracking estrous cycles during assessment or phase-locking assessments to one estrous stage would better elucidate the effects of hormones on motivated behavior and potentially reduce variability.

Though this study did not directly observe the effects of MJN110 against HAND in terms of behavioral outcomes, it provides insights into its potential for neuroprotection and as a modulator of neuroinflammatory responses in the frontostriatal circuitry. The MJN110 observed increase of Tat-induced downregulation of MAP2ab^+^ dendritic intensity and reduction of neuroinflammatory markers demonstrates promise for curbing the underlying inflammatory priming linked with HIV-1, potentially diminishing the emergence or persistence of additional behavioral sequelae associated with HAND. To better clarify the involvement of other cytokines in these effects, further quantification of TNF-α would be advantageous. Because TNF-α inhibits astrocytic glutamate uptake and increases reactive oxygen species (ROS) (98, 99), characterizing HIV-1-associated effects on this measure would enable elucidation of additional mechanisms by which these cells become dysfunctional in infection and may be restored with MJN110. Notably, biomarkers of oxidative stress are elevated in PLWH treated with highly active antiretroviral drugs (100). This increased oxidative stress contributes to neurotoxicity, HAND, and premature aging (100, 101). The observed upregulation of MAP2ab^+^ dendritic signal by MJN110 in Tat(+) mice and MJN110-induced reduction in ROS-derived 11-HETE in the present study suggests a mechanism apart from other enzymatic pathways by which this treatment may be neuroprotective, and collecting data for TNF-α would provide insight into whether the reduction in oxidative stress biomarkers is related to possible additional MJN110-driven immunoreactivity-suppressing effects. Assessing lipid mediator expression in the infralimbic cortex would also provide valuable data to determine whether the effects of Tat and MJN110 may be distinct from those observed in the striatum. As hippocampal lipid mediator expression analyses revealed Tat-induced upregulation of 11-HETE (**Supplementary Figure S1D**), additional region-specific effects may also be observed elsewhere. Given the differences in neuroinflammatory phenotypes between the infralimbic cortex and striatum in astrocyte density and colocalization with IL-1β, it remains possible that diverse cellular mechanisms are recruited to drive distinct inflammatory responses across regions.

In contrast to previous studies of MAGL inhibition, which have shown reductions in prostaglandin expression (102), no such alteration was observed in our analyses (see **Figure 6** and **Supplementary Figure S1** heatmap panel sections for COX pathway). However, the underlying difference may involve which specific MAGL inhibitor is administered, what dosage is used, or a combination of the two factors. MJN110 is a newer-generation drug relative to those used in prior work, with benefits over previous-generation drugs as described earlier, including fewer off-target effects. Despite the similarity in that prostaglandins and HETEs are both inflammatory metabolites of arachidonic acid (AA), their derived mechanisms differ in their metabolizing enzyme. Prostaglandins are derived from cyclooxygenase (COX), whereas HETEs are largely derived from lipoxygenases (LOX) and cytochrome P450 (CYP) enzymes (103). That MJN110 decreased HETEs without affecting prostaglandins suggests a more COX-independent pathway for MJN110’s profile of anti-inflammatory effects. This finding is important because while COX-targeting therapeutics such as non-steroidal anti-inflammatory drugs (NSAIDs) are effective against inflammation and pain, they can also increase the risk of adverse cardiovascular events (104–106). MJN110 confers anti-inflammatory effects while more specifically targeting pathways beneficial to cardiac, respiratory, and cerebrovascular health, including LOX and CYP (107, 108), with the exception of 5-HETE, which is derived from AA through the LOX system but metabolized through the COX system (109). Given predispositions of PLWH to cardiovascular disease and related complications (110, 111), this therapeutic strategy may improve quality of life across more domains relative to earlier-developed MAGL inhibitors, which interact with COX pathways. Of note, HIV-1 gp120 is more strongly linked to increases in signaling within the COX pathway, particularly through induction of COX-2 expression (112), so using subjects who express more constituent proteins of the HIV-1 viral genome may provide a more complete understanding of virus-associated inflammatory profiles as well as therapeutic potential of MJN110 across signaling pathways.

Within astrocytic mechanisms, assessing other factors, such as neurotrophin synthesis and hypertrophy, could further elucidate additional contrasting effects of Tat and MJN110 on astrogliosis. Astrocyte density was assessed presently as increased astrocyte counts are widely understood to indicate increased proliferation associated with astrogliosis (113); however, a limitation remains in the inability to distinguish between normal and activated astrocytes. There also remains the question of other cellular mechanisms potentially driving independent inflammatory responses. While number of microglia and microglia morphology was not altered by Tat and MJN110, microglia are recognized as a productive viral reservoir that contributes to IL-1β upregulation through stimulation of nucleotide-binding domain leucine-rich repeat-containing proteins (NLRs) (114). The present study could not further probe microglial activation-induced IL-1β upregulation due to technical difficulties with triple-immunolabeling, but these contributions to inflammatory tone remain important to consider.

While the expression and distribution of MAGL were not assessed in the current study, recently published work specifically examined MAGL expression in our transgenic mouse model, and demonstrated Tat induction does not alter MAGL expression (87). Based on these findings, it can reasonably be inferred that MAGL expression was not a confounding factor in the data observed presently. Additionally, a previous paper authored by our lab provides relevant insights into MJN110’s effects in the brain (62). This study, which utilized the same subject preparation methods, including dosage, frequency of MJN110 administration, and duration of Tat induction as the present study, investigated the effect of MJN110 on endocannabinoid levels in various brain regions and showed that treatment with MJN110 increased levels of 2-AG in multiple brain regions. These findings suggest that this MAGL inhibitor effectively modulated endocannabinoid signaling in the brain without baseline MAGL alteration driven by Tat. Nevertheless, to directly conclude that MJN110 effects are due to increases in 2-AG levels, mice should be administered a 2-AG synthesis blocker, such as DO34, similar to what has been published previously (60).

Several limitations of this study should be considered when applying current findings to the therapeutic potential of targeting MAGL in the context of HIV. First, despite the Tat transgenic mouse model being a well-established neuroHIV model, only one of the many viral proteins is expressed. It is known that viral proteins can interact and target various signaling pathways (115); thus they can modify CNS and behavior in different ways compared to a single viral protein. Second, the use of doxycycline for inducing Tat expression is not ideal, as doxycycline has neuroprotective effects on its own (116). Nevertheless, both Tat(–) and Tat(+) mouse groups received doxycycline chow throughout the study to control for this confound and minimize bias. Third, only females were used in the current study without monitoring estrous cycle. Future studies should monitor estrous cycle as hormones can alter motivated behavior associated with a food reward (94), as well as include male mice for comparison, especially due to the known sex-specific differences in immune response to the virus (117). Lastly, ART medication was not considered in the study, as the HIV Tat transgenic mouse model mimics individuals on ART because no virus replication/entry/integration is present, similar to what is seen in PLWH on ART with undetectable viral load. Nevertheless, it is known that cannabinoids and ART drugs are metabolized through the CYP450 system, thus leading to potential drug-drug interaction (118). Interestingly, no significant interactions have been reported between cannabinoids and HIV protease inhibitors in previous studies (119, 120), but additional pharmacokinetic studies remain necessary to increase our understanding of cannabinoid-ART interactions.

## 5 Conclusion

A wide body of evidence indicates while HIV-1 treatment has improved life expectancy and quality over the years, supplemental strategies and studies are needed to address persistent issues with patient health, shortcomings of cART, and historically problematic research design. Ultimately, understanding the specific virus-associated vulnerabilities across different subpopulations – and especially the therapeutic potential of novel adaptogenic compounds like MJN110, as demonstrated here – will enhance the foundation of knowledge upon which similar future studies are built and improve management strategies for HIV-1 and other inflammatory diseases.

## Data availability statement

The raw data supporting the conclusions of this article will be made available by the authors, without undue reservation.

## Ethics statement

The animal study was approved by University of North Carolina at Chapel Hill Institutional Animal Care and Use Committee. The study was conducted in accordance with the local legislation and institutional requirements.

## Author contributions

AFL: Writing – review & editing, Writing – original draft, Visualization, Validation, Project administration, Methodology, Investigation, Funding acquisition, Formal analysis, Data curation, Conceptualization. BJY-S: Formal analysis, Data curation, Writing – review & editing. RK: Data curation, Writing – review & editing, Investigation. CAC: Investigation, Formal analysis, Writing – review & editing. IRJ: Methodology, Writing – review & editing. JM: Writing – original draft, Validation, Formal analysis, Data curation, Writing – review & editing, Resources. MJN: Writing – review & editing, Resources. BFC: Writing – review & editing, Resources. AHL: Writing – review & editing, Resources. BMI-J: Writing – review & editing, Resources. SF: Writing – review & editing, Writing – original draft, Validation, Supervision, Resources, Project administration, Methodology, Investigation, Funding acquisition, Formal analysis, Conceptualization.
