# Supplementary material for: A helping HAND: therapeutic potential of MAGL inhibition against HIV-1-associated neuroinflammation
Source: Front Immunol. 2024 May 21;15:1374301. doi: 10.3389/fimmu.2024.1374301 (PMC11148243; doi:10.3389/fimmu.2024.1374301)
Supplement: Supplementary file 1 [file Presentation_1.pdf]

A Helping HAND: Therapeutic Potential of MAGL Inhibition Against HIV-1-associated Neuroinflammation

Alexis F. League[1,*], Barkha J. Yadav-Samudrala[1], Ramya Kolagani[1], Calista A. Cline[1], Ian R. Jacobs[1], Jonathan Manke[2], Micah J. Niphakis[3], Benjamin F. Cravatt[3], Aron H. Lichtman[4], Bogna M. Ignatowska-Jankowska[5], and Sylvia Fitting[1,*]

[1] Department of Psychology and Neuroscience, University of North Carolina at Chapel Hill, Chapel Hill, NC 27599, USA
[2] Department of Pharmaceutical Sciences, Skaggs School of Pharmacy and Pharmaceutical Sciences, University of Colorado Anschutz Medical Campus, Aurora, CO 80045, USA
[3] Department of Chemistry, Scripps Research, La Jolla, CA 92037, USA
[4] Department of Pharmacology and Toxicology, Virginia Commonwealth University, Richmond, VA 23284, USA
[5] Neuronal Rhythms in Movement Unit, Okinawa Institute of Science and Technology, Okinawa, 904-0495, JP
* Correspondence: leagueaf@protonmail.com, sfitting@email.unc.edu

Keywords: HIV-1; inflammation; transactivator of transcription (Tat); endocannabinoids; HETE; monoacylglycerol lipase (MAGL)

Supplemental Material

Figure Caption

Figure S1. Quantification of hippocampal lipid mediators. Heatmap of all lipid mediators assessed (**top**). Summary data from significant findings (**A-D**). (**A**) A significant genotype x drug interaction was noted for 12-HETE with group comparisons revealing no significant differences. (**B, C**) MJN110 treatment significantly decreased 15-HETE and 5-HETE/14(15)-EET levels. (**D**) Tat expression significantly increased 11-HETE levels, which was specifically seen in the vehicle-exposed groups, also demonstrated by a significant genotype x drug interaction. All data are expressed as mean ± the standard error of the mean (SEM). Statistical significance was assessed by ANOVAs followed by Tukey's post hoc tests when appropriate; $^{*}p = 0.046$ main effect of genotype, $^{\#}p < 0.05$ main effect of drug, $^{\S}p \leq 0.05$ genotype x drug interaction, $^{a}p = 0.032$ vs. vehicle-treated Tat(+) mice. Individual subject data are represented by open circles.

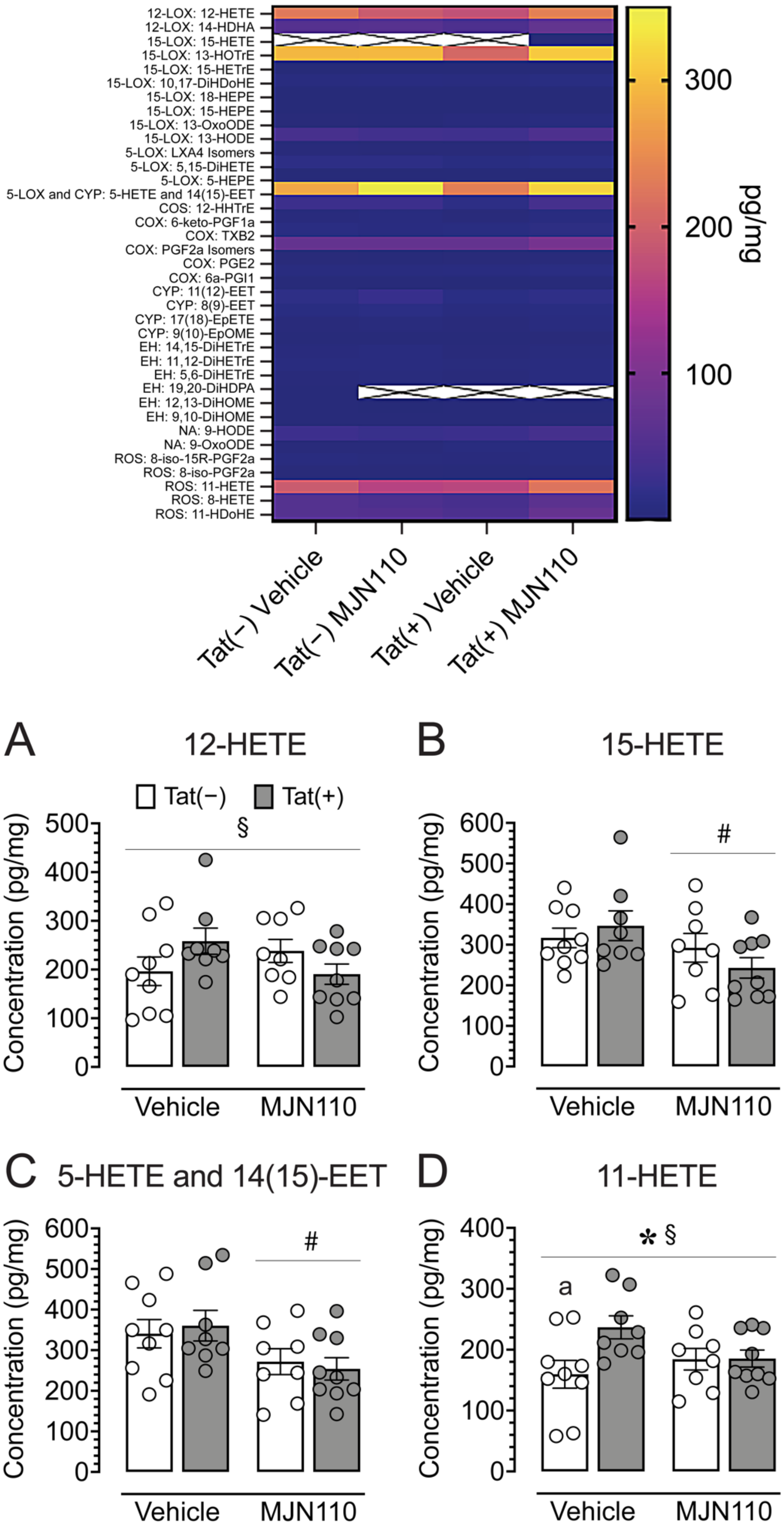
12-LOX: 12-HETE
12-LOX: 14-HDHA
15-LOX: 15-HETE
15-LOX: 13-HOTrE
15-LOX: 15-HETrE
15-LOX: 10,17-DiHDoHE
15-LOX: 18-HEPE
15-LOX: 15-HEPE
15-LOX: 13-OxoODE
15-LOX: 13-HODE
5-LOX: LXA4 Isomers
5-LOX: 5,15-DiHETE
5-LOX: 5-HEPE
5-LOX and CYP: 5-HETE and 14(15)-EET
COS: 12-HHTrE
COX: 6-keto-PGF1a
COX: TXB2
COX: PGF2a Isomers
COX: PGE2
COX: 6a-PGI1
CYP: 11(12)-EET
CYP: 8(9)-EET
CYP: 17(18)-EpETE
CYP: 9(10)-EpOME
EH: 14,15-DiHETrE
EH: 11,12-DiHETrE
EH: 5,6-DiHETrE
EH: 19,20-DiHDPA
EH: 12,13-DiHOME
EH: 9,10-DiHOME
NA: 9-HODE
NA: 9-OxoODE
ROS: 8-iso-15R-PGF2a
ROS: 8-iso-PGF2a
ROS: 11-HETE
ROS: 8-HETE
ROS: 11-HDoHE
300
200
100
pg/mg
Tat(−) Vehicle
Tat(−) MJN110
Tat(+) Vehicle
Tat(+) MJN110
A
12-HETE
Tat(−)
Tat(+)
§
Concentration (pg/mg)
500
400
300
200
100
0
Vehicle
MJN110
B
15-HETE
#
Concentration (pg/mg)
600
500
400
300
200
100
0
Vehicle
MJN110
C
5-HETE and 14(15)-EET
#
Concentration (pg/mg)
600
500
400
300
200
100
0
Vehicle
MJN110
D
11-HETE
* §
a
Concentration (pg/mg)
400
300
200
100
0
Vehicle
MJN110
